# Individual CpG sites that are associated with age and life expectancy become hypomethylated upon aging

**DOI:** 10.1186/s13148-017-0315-9

**Published:** 2017-02-02

**Authors:** Yan Zhang, Jan Hapala, Hermann Brenner, Wolfgang Wagner

**Affiliations:** 1 Division of Clinical Epidemiology and Aging Research, German Cancer Research Center (DKFZ), Im Neuenheimer Feld 581/TP4, 69120 Heidelberg, Germany; 2 Helmholtz-Institute for Biomedical Engineering, Stem Cell Biology and Cellular Engineering, University Hospital of the RWTH Aachen, Pauwelsstrasse 20, 52074 Aachen, Germany; 3 Institute for Biomedical Engineering—Cell Biology, University Hospital of the RWTH Aachen, Pauwelsstrasse 30, 52074 Aachen, Germany; 4 Network Aging Research (NAR), University of Heidelberg, Bergheimer Strasse 20, 69120 Heidelberg, Germany

**Keywords:** DNA methylation, Epigenetic, Aging, Mortality, Life expectancy, Predictor

## Abstract

**Background:**

There is a growing interest in simple molecular biomarkers for biological aging. Age-associated DNA methylation (DNAm) changes at specific CG dinucleotides can be combined into epigenetic age predictors to estimate chronological age—and the deviation of chronological and predicted age (∆_age_) seems to be associated with all-cause mortality. In this study, we have further validated this association and analyzed whether or not individual age-associated CG-dinucleotides (CpGs) are related to life expectancy.

**Findings:**

In the German ESTHER cohort, we used 864 DNAm profiles of blood samples as the discovery set and 1000 DNAm profiles as the validation set to predict chronological age with three previously reported age predictors—based on 99, 71, or 353 age-associated CpGs. Several of these individual CpGs were significantly associated with life expectancy, and for some of these CpGs, this was even reproducible in the independent datasets. Notably, those CpGs that revealed significant association with life expectancy were overall rather hypomethylated upon aging.

**Conclusion:**

Individual age-associated CpGs may provide biomarkers for all-cause mortality—but confounding factors need to be critically taken into consideration, and alternative methods, which facilitate more quantitative measurements at individual CpGs, might be advantageous. Our data suggest that particularly specific CpGs that become hypomethylated upon aging are indicative of biological aging.

**Electronic supplementary material:**

The online version of this article (doi:10.1186/s13148-017-0315-9) contains supplementary material, which is available to authorized users.

## Findings

Biomarkers for aging may allow for testing of interventions to extend lifespan or to increase the odds of staying healthy. Ideally, such biomarkers should rather reflect “biological age” than “chronological age,” and they should not be skewed by predisposition to specific diseases [1]. Advances in molecular biology, genetics, and epigenetics have fueled the hope for simple and reliable biomarkers for biological age [2, 3].

Within the last five years, a multitude of studies demonstrated that aging is associated with highly reproducible DNA methylation (DNAm) changes at specific sites in the genome [4–8]. About 60% of these age-associated CG dinucleotides—so called “CpG sites”—become hypomethylated upon aging, whereas about 40% become hypermethylated [9]. Age-associated hypermethylation is rather enriched close to CG islands (CGIs), whereas hypomethylation rather occurs outside of CGIs [9–12]. Furthermore, particularly DNAm at CpGs with age-associated hypermethylation seem to be coherently modified in cancer [13], indicating that de novo DNAm and demethylation may be regulated by different mechanisms. It is yet unclear how these DNAm patterns are regulated, and if they are functionally relevant or rather reflect other means of chromatin conformation—either way, they provide powerful biomarkers.

Several age-associated DNAm changes are acquired linearly over time and hence facilitate estimation of chronological age—either based on individual CpGs [14] or by integration of multiple CpGs into age predictors [5, 6, 12]. Particularly, the epigenetic clock described by Horvath [15], consisting of 353 age-associated CpGs, has been shown to facilitate precise age estimations across multiple tissues. Other frequently used age predictors for blood samples have been introduced by Hannum and coworkers (71 CpGs) [16] and Weidner et al. (99 CpGs) [17, 18]. Notably, the difference between chronological age and predicted age—referred to as ∆_age_—seems to be related to the parameters of biological aging: Marioni and coworkers have demonstrated that ∆_age_ (per 5 years) was associated with a 21% higher mortality risk in the “Hannum predictor” (95% CI 1.14–1.29) and with a 11% higher mortality risk with the “Horvath predictor” (95% CI 1.05–1.18), if adjusted for chronological age and gender [19]. Similar findings were reproduced by other study groups on other datasets [18, 20, 21]. Furthermore, epigenetic age predictions are lower in women and in semi-supercentenarians [22], whereas accelerated epigenetic age was associated with obesity [23] and with lower abilities in physical and mental fitness [24]—suggesting that age-associated DNAm patterns may be indicative of biological aging.

In this study, we aimed for a better understanding of how epigenetic age predictions are associated with life expectancy in the ESTHER study cohort, a large population-based epidemiological study conducted in the German State of Saarland. To estimate reproducibility of results, we separated the DNAm profiles (analyzed by HumanMethylation 450 BeadChips) into a discovery set of 864 samples and a validation set of 1000 samples (further information is provided in the Additional file 1). We were particularly interested whether there are individual CpGs that reveal higher association with life expectancy than others.

### Comparison of different multi-CpG age predictors

**Fig. 1:**
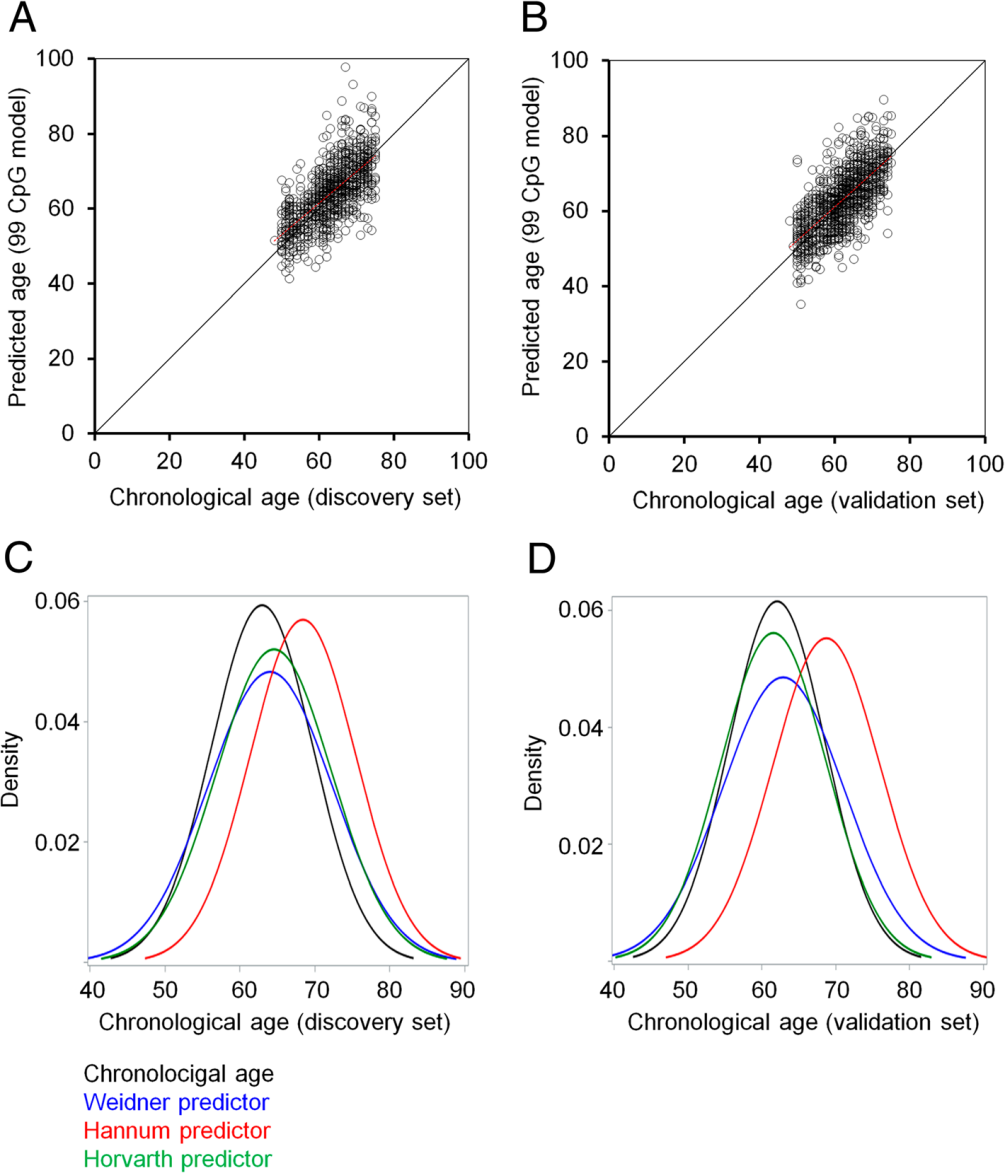
Correlation of predicted age with chronological age. Epigenetic age predictions based on the 99 CpGs of the Weidner predictor [17] were plotted against chronological age for **a** 864 DNAm profiles of the discovery set and **b** 1000 DNAm profiles of the validation set of the ESTHER cohort. The distribution of chronological age and predicted age with the three aging models described by Weidner et al. [17], Hannum et al. [16], and Horvath [15] is demonstrated **c** for the discovery set and **d** for the validation set. Age predictions by the Hannum predictor were overall overestimated by 5.5 and 6.5 years, respectively

Initially, we compared epigenetic age predictions of the three aging models by Horvath [15], Hannum et al. [16], and Weidner et al. [17] in the discovery and validation sets, as well as in the overall population (Table 1). Overall, all three models revealed good correlation with chronological age, albeit the correlation was slightly lower for the Weidner model (Fig. 1a, b). On the other hand, epigenetic age predictions of the Hannum predictor were on average overestimated by 5.5 years in the discovery set and 6.5 years in the validation set (Fig. 1c, d). Hence, the mean average deviation (MAD) of predicted and chronological age was higher for the Hannum predictor in the discovery and validation set than for the other two predictors (Table 1). Such shifts do not affect inter-quartile comparison, Cox regression analysis, or hazard ratios, which are usually described in the literature. However, they have impact on ∆_age_ and should therefore be taken into consideration if ∆_age_ is addressed for individual patients or for direct comparison of different datasets. It is conceivable that the higher MAD in one or the other epigenetic age predictor is due to prevalence of specific diseases. “Healthy subjects” are difficult to define, and therefore, we have exemplarily excluded participants with prevalent diabetes, cardiovascular disease, and a history of cancer at baseline (discovery panel: 180, 189, and 75, respectively; validation set: 162, 182, and 66, respectively). Removal of these participants resulted in a very similar distribution of age predictions, indicating that general offset of the age predictors was not due to these chronic diseases (Additional file 1: Figure S1).

**Table 1:** Correlation of age predictions with chronological age

	Weidner99 CpGs (61 hypo- and 38 hypermethylated)	Hannum71 CpGs (31 hypo- and 40 hypermethylated)	Horvath 353 CpGs (186 hypo- and 167 hypermethylated)
Discovery set (*n* = 864)			
Correlation with age (Spearman)	0.705	0.809	0.761
Mean average deviation (years)	4.76	5.82	4.19
Validation set (*n* = 1000)			
Correlation with age (Spearman)	0.712	0.774	0.750
Mean average deviation (years)	4.78	7.00	3.95
Overall (*n* = 1864)			
Correlation with age (Spearman)	0.705	0.787	0.753
Mean average deviation (years)	4.75	6.45	4.06

Previous studies have demonstrated that ∆_age_ of the Hannum and Horvath predictors are associated with life expectancy in DNAm profiles of the ESTHER study [20]. Here, we have analyzed if ∆_age_ of the Weidner model would also be associated with all-cause mortality. When the results were adjusted for age, sex, batch, and leucocyte distribution, there was a clear tendency in the discovery and validation sets, but the results did not reach statistical significance (*P* = 0.058 and *P* = 0.095, respectively). When we combined the discovery and validation sets to increase statistical power, the results reached the significance (*P* = 0.041) and the hazard ratios were slightly lower than in the other two predictors (HR = 1.087; 95% CI 1.003–1.178; Additional file 1: Table S1). In our previous work, we analyzed the data of the Lothian Birth Cohort 1921 (LBC1921), a study from the Lothian region (Edinburgh and its surrounding areas of Scotland) with participants born in 1921 and analyzed at about the age of 79 [18, 25]: in this dataset a 5-year higher age prediction by the Weidner model was associated with 11% greater mortality risk (*P* = 0.0003; 95% CI 1.04, 1.19; after adjustment for age and gender). These results support the notion that the association of ∆_age_ with all-cause mortality may vary between different aging models and cohorts—but it is overall consistent if using age predictors that comprise multiple CpGs.

### Individual CpGs are associated with life expectancy

We have previously analyzed if individual age-associated CpGs are associated with life expectancy in the Lothian Birth Cohorts 1921 and 1936 [18]. The only one CpG site that reached statistical significance in both datasets after multiple correction and adjustment for age and gender was cg05228408, which is associated with the gene for the chloride transport protein 6 (*CLCN6*; LBC1921 [HR = 1.16; 95% CI 1.06–1.26; *P* = 0.00072]; LBC1936 [HR = 1.26; 95% CI 1.12–1.42; *P* = 0.00013]). This genomic region is of specific interest because single-nucleotide polymorphisms identified in its vicinity were found to be associated with blood pressure and hypertension [26–28]. Therefore, we have now trained a model for the ESTHER discovery group based on the beta values of cg05228408. Upon the adjustment for chronological age, gender, batch, and leucocyte distribution, this model revealed significant association with all-cause mortality in the discovery (*P* = 0.0011) and in the overall population (*P* = 0.0148; Additional file 1: Table S2).

**Fig. 2:**
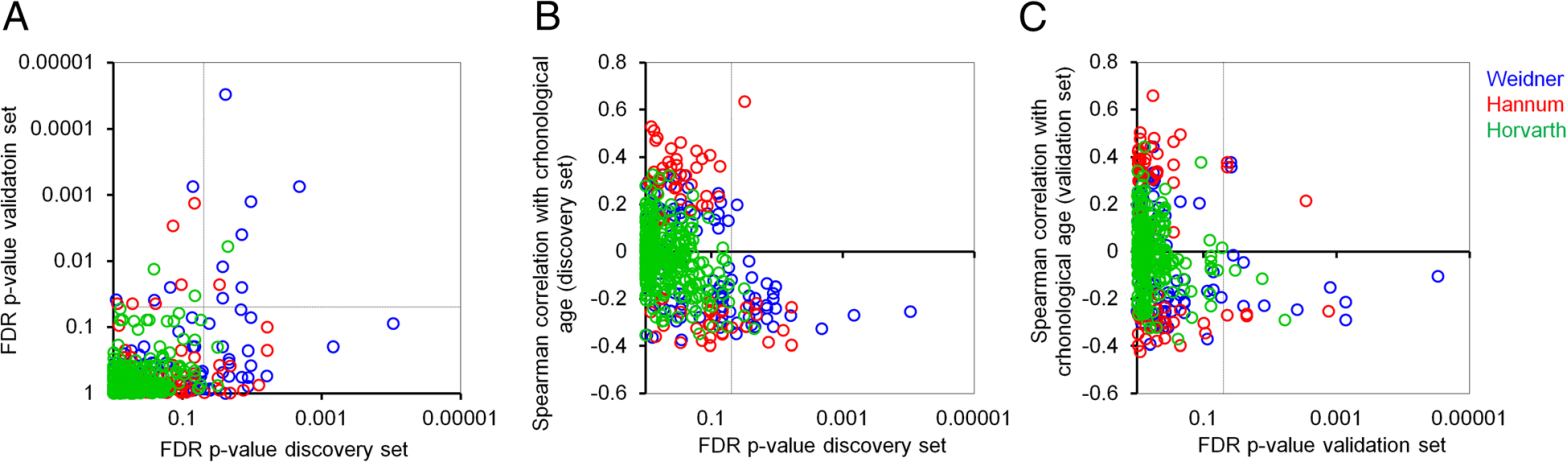
CpGs that correlate with all-cause mortality are hypomethylated upon aging. **a** For all individual CpGs of the three age predictors (Weidner et al., 99 CpGs; Hannum et al., 71 CpGs; and Horvath, 353 CpGs), the association of ∆_age_ with all-cause mortality was estimated. The *P* values in the discovery and validation sets of the ESTHER cohort demonstrate moderate reproducibility between the two independent datasets. **b**, **c** Subsequently, we analyzed the Spearman correlation of these CpGs with chronological age. CpGs with significant association with all-cause mortality were overall hypomethylated upon aging (in the discovery set (**b**) and in the validation set (**c**)). The lines indicate a FDR significance level of 0.05

Subsequently, we tested the association with life expectancy for all individual CpGs of the three age predictors: for 99 CpGs of the Weidner predictor (Additional file 1: Table S3), for 71 CpGs of the Hannum predictor (Additional file 1: Table S4), and for the 353 CpGs of the Horvath predictor (Additional file 1: Table S5). In the discovery set, 27 (of 99 CpGs), 11 (of 71 CpGs), and 3 CpGs (of 353 CpGs) reached statistical significance (FDR < 0.05). In the validation set, with a lower number of death cases, it was only 11, 7, and 3 CpGs, respectively (Fig. 2a). Albeit the reproducibility between the two datasets was not very high, there was a significant association for the 99 CpGs of the Weidner predictor (hypergeometric distribution: *P* value = 0.0072) and for the Horvath predictor (*P* value = 0.025; Additional file 1: Table S6). The CpGs that were overlapping associated with life expectancy in both datasets were cg05294455 (*MYL4*), cg08598221 (*SNTB1*), cg09462576 (*MRPL55*), cg15804973 (*MAP3K5*), cg20654468 (*LPXN*), cg25268718 (*PSME1*), cg26581729 (*NPDC1*), and cg02867102 (no gene). Please note that the number of individual CpGs that reached statistical significance in the three predictors is not a quality measure for these age predictors. The CpGs of the Hannum and Horvath predictors were selected by Elastic Net algorithms—they were therefore selected to work together, rather than individually. Furthermore, the Horvath predictor was trained on multiple tissues rather than blood samples as in the Hannum and Weidner predictors.

To our surprise, almost all of the CpGs that are associated with life expectancy in either of the two datasets were hypomethylated upon aging (Fig. 2b, c). In the discovery set there was a significant enrichment of hypomethylated CpG sites (hypergeometric distribution) for the Weidner (*P* = 3.3 × 10^−6^) and the Hannum *(P* = 0.0007) predictor. Furthermore, all significant CpGs in the overlap of the discovery and the validation set were hypomethylated (Additional file 1: Table S6).

We revisited the previously published data on association of these CpGs in the Lothian Birth Cohort 1921 [18]. A big advantage in this cohort is that it comprises donors of a defined age range (about 79 years)—and hence, a different slope in the comparison of predicted and chronological ages would hardly affect the association with life expectancy. Only four CpGs of the Weidner predictor reached statistical significance in LBC1921 (adjusted *P* value <0.05), and all of them were also significant in the ESTHER discovery set: cg05228408 (*CLCN6*), cg12554573 (*PARP3*), cg25268718 (*PSME1*), and cg03224418 (*SAMD10*)—furthermore, all of them become hypomethylated upon aging (Additional file 1: Figure S2A). However, for the CpGs of the Hannum predictor, the reproducibility between the LBC1921 and the ESTHER cohorts was low. In general, CpGs that revealed significant association with life expectancy in LBC1921 and LBC1936 were rather hypomethylated, but these results did not reach statistical significance (Additional file 1: Figure S2B, C).

## Conclusions

Our explorative study further supports the notion that specific age-associated CpGs can be indicative of life expectancy, but the reproducibility in independent cohorts is overall not very high. Furthermore, we demonstrate that significant association with all-cause mortality is particularly observed in CpGs that become hypomethylated upon aging. It is therefore conceivable that a combination of such specific age-associated CpGs gives rise to alternative epigenetic age predictors that better reflect the association of ∆_age_ with all-cause mortality—and may hence be a better biomarker for biological aging.

There are however limitations that need to be critically taken into consideration: (1) only blood samples have been considered for this analysis, and it remains to be demonstrated if the findings hold also true for cells from other tissues; (2) the association of life expectancy with CpGs that become hypomethylated upon aging was only addressed on elderly people, whereas biomarkers for biological aging may rather be desired for young humans who had not yet developed age-related diseases [29]; (3) ∆_age_ of epigenetic age predictions may have systematic offsets, and hence, it remains a challenge to entirely rule out that the results are impacted by chronological age; (4) the beta values of Illumina BeadChip correlate with the absolute level of DNAm, but the precision is not always high [30]. Particularly, for age predictors based on individual CpGs, it therefore appears to be advantageous to train model on data that was generated by more quantitative methods—such as pyrosequencing, MassARRAY, bisulfite deep sequencing, or digital PCR [18]; and (5) last but not least, the association with all-cause mortality is only one aspect of biological aging, and it will be important to better understand the association with other molecular parameters, such as telomere length, or functional measures, such as physical strength, cognitive decline, and other signs of aging [3].

## Additional file

Additional file 1: This file contains additional details on the methods, Additional file 1: Figures S1–S2, and Additional file 1: Tables S1–S6. (PDF 1054 kb)
