# Supplementary material for: Individual CpG sites that are associated with age and life expectancy become hypomethylated upon aging
Source: Clin Epigenetics. 2017 Feb 2;9:9. doi: 10.1186/s13148-017-0315-9 (PMC5288846; doi:10.1186/s13148-017-0315-9)
Supplement: Additional file 1: — This file contains additional details on the methods, Additional file 1: Figures S1–S2, and Additional file 1: Tables S1–S6. (PDF 1054 kb) [file 13148_2017_315_MOESM1_ESM.pdf]

Supplemental Data

Individual CpG Sites that are Associated with Age and Life Expectancy become Hypomethylated upon Aging

Yan Zhang[1], Jan Hapala[2,3], Hermann Brenner[1,4], and Wolfgang Wagner[2,3]

[1] Division of Clinical Epidemiology and Aging research, German Cancer Research Center (DKFZ), Im Neuenheimer Feld 581/TP4, 69120 Heidelberg, Germany; [2] Helmholtz-Institute for Biomedical Engineering, Stem Cell Biology and Cellular Engineering, University Hospital of the RWTH Aachen, Pauwelsstrasse 20, 52074 Aachen, Germany; [3] Institute for Biomedical Engineering - Cell Biology, University Hospital of the RWTH Aachen, Pauwelsstrasse 30, 52074 Aachen, Germany; [4] Network Aging Research (NAR), University of Heidelberg, Bergheimer Strasse 20, 69120 Heidelberg, Germany

Table of Content

Methods 2

Study population 2
DNA methylation measurements 2
Derivation of age predictors 2
Survival analysis and statistics 3

Supplemental figures 3

Figure S1. Predicted ages in participants without diabetes, cardiovascular disease, and cancer. 3
Figure S2. Reanalysis of life-expectancy associated CpGs in the LBC1921 and LBC1936 cohorts. 4

Supplemental tables 5

Supplemental table S1. Association of Weidner model with all-cause mortality. 5
Supplemental table S2. Association of cg05228408 (*CLCN6*) with all-cause mortality. 5
Supplemental table S3: Associations of Weidner-CpGs with all-cause mortality. 6
Supplemental table S4: Association of Hannum CpGs with all-cause mortality 8
Supplemental table S5: Association of Horvath-CpGs with all-cause mortality. 9
Supplemental table S6: Overlap of significant CpGs in discovery and validation set. 16

References of supplemental data 16

Methods

Study population

The ESTHER cohort is a large population-based epidemiological study, in which general practitioners (GP) recruited 9,949 adults (age 50 - 75 years) during regular health check-ups in the German state of Saarland between 2000 and 2002, with the aim of assessing chances of prevention and early detection of chronic diseases [1]. All participants provided written informed consent. The study was approved by the ethics committees of the University of Heidelberg and of the state medical board of Saarland, Germany.

Two subsets of ESTHER participants were selected for genome-wide DNAm analysis:
The discovery set, based on a case-cohort design, included 406 participants, who died during follow-up by March 2013 among 2,499 ESTHER participants recruited between October 2000 and March 2001; furthermore 548 participants were randomly selected among the 2,499 (as the subcohort in the case-cohort samples) irrespective of death status during follow-up. Ninety of 406 deaths were also included in the subcohort owing to random selection of subcohort at baseline, and the discovery set therefore includes total of 864 participants.

The validation set consists of 1,000 ESTHER participants who were recruited during the initial enrollment (between July and October 2000) and who were non-overlapping with the case-cohort samples. Of the 1,000 participants, 231 deaths were ascertained during follow-up. Details of study design and data collection were described before [2].

At baseline, all participants completed a standardized self-administered questionnaire and donated biological samples (blood, stool, urine). Comprehensive medical data, such as the results of a physical assessment, medical diagnoses, and drug prescriptions were additionally obtained from the GPs. Prevalent diabetes was defined by physician diagnosis or the use of glucose-lowering drugs. Prevalent cardiovascular disease (CVD) at baseline was defined by either physician-reported coronary heart disease or a self-reported history of a major cardiovascular event, such as myocardial infarction, stroke, pulmonary embolism or revascularization of coronary arteries. Prevalent cancer [ICD-10 codes C00-C99; non-melanoma skin cancer (C44) was excluded] was ascertained by self-report or record linkage with data from the Saarland Cancer Registry (http://www.krebsregister.saarland.de /ziele/ziel1.html).

DNA methylation measurements

Genomic DNA of blood was extracted using a salting out procedure [3] and DNAm profiles were analyzed using the Infinium HumanMethylation450 BeadChip (Illumina Inc.) in the Genomics and Proteomics Core Facility at the German Cancer Research Center, Heidelberg, Germany. For each of about 480,000 CpGs the DNAm levels were calculated with Illumina's Genomestudio 2011.1, Module M version 1.9.0 as previously described in detail [4]. Data were normalized to internal controls provided by Illumina. In data pre-processing, probes with detection p-value>0.05 and with missing values>10% were excluded.

Derivation of age predictors

The epigenetic age-predictors by Hannum and coworkers [5] and Horvath [6] were calculated as described before [2]. The 99-CpG model, i.e. "Weidner predictor", was initially derived from 102 CpGs that revealed linear age-associated changes in 575 DNAm profiles of blood that were generated on Illumina HumanMethylation27 BeadChips (Pearson correlation $R > 0.85$ or $R < -0.85$; age range 0 to 78 years) as described in detail before [7, 8].

Survival analysis and statistics

The associations of Δ_{age} or of individual CpGs with all-cause mortality were analyzed by weighted Cox regression models that account for the case-cohort sampling design in the discovery set [9, 10], and by multiple Cox regression in the validation set. The models with Δ_{age} or methylation β-values of each CpGs as explanatory variables were adjusted for age (continuous) and sex only (model-I); and additionally adjusted for batch effect, and leukocyte composition estimated by Houseman's algorithm [11] (model-II). Multiple testing was corrected for by the Benjamini-Hochberg approach [False Discovery Rate (FDR) < 0.05]. The statistical analyses were performed in SAS 9.4 (SAS Institute, Cary, NC). The probability of sampling distribution of significant CpGs was estimated by hypergeometric distribution.

Supplemental figures

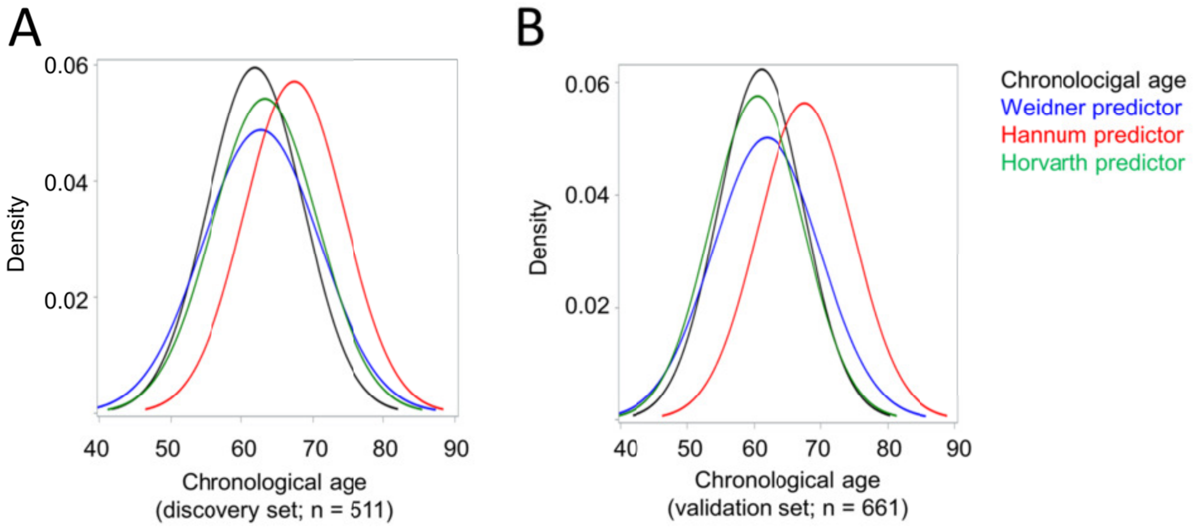

Figure S1. Predicted ages in participants without diabetes, cardiovascular disease, and cancer. These density distribution curves only comprise participants without the above mentioned chronic diseases in the discovery set (A; 511 participants) and validation set (B; 661 participants) at baseline. Please note that the results look similar to the distributions that comprise the participants with known prevalent diabetes, CVD and a history of cancer at baseline (Figure 1C/D).

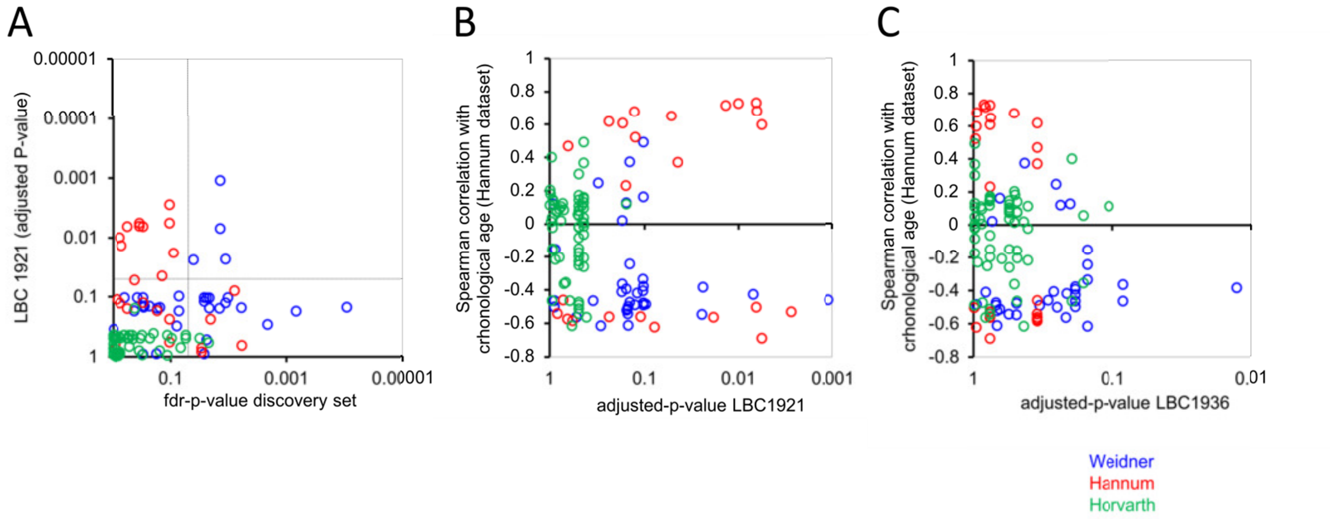

Figure S2. Reanalysis of life-expectancy associated CpGs in the LBC1921 and LBC1936 cohorts. In our previous study, we have tested for association of individual CpGs of the three age-predictors with mortality in the Lothian Birth Cohorts 1921 and 1936 [7]. (A) These data were now correlated with the p-values of the discovery set of the ESTHER cohort. All four CpGs of the Weidner predictor that revealed significant results in LBC1921 were also significant in the ESTHER discovery set (p-value = 0.0047). However, the reproducibility between the different datasets was overall rather moderate. (B,C) The Lothian Birth Cohorts have only a very small range of donor-age and therefore we have used the datasets described by Hannum and coworkers [5] to estimate if the CpGs are rather hypermethylated (positive Spearman correlation) or hypomethylated upon aging (negative Spearman correlation). In tendency, significant association with life-expectancy was rather observed in hypomethylated CpGs.

Supplemental tables

Supplemental table S1. Association of Weidner model with all-cause mortality.

Parameter	Hazard ratio	95% CI	p-value
Discovery set (n = 864)			
Weidner predictor	1.120	[0.996; 1.261]	0.058
Age	1.082	[1.056; 1.107]	<.0001
Sex	0.461	[0.339; 0.627]	<.0001
Validation set (n = 1000)			
Weidner predictor	1.091	[0.985; 1.208]	0.0949
Age	1.118	[1.092; 1.144]	<.0001
Sex	0.542	[0.406; 0.725]	<.0001
Overall (n = 1864)			
Weidner predictor	1.087	[1.003; 1.178]	**0.0412**
Age	1.091	[1.072; 1.110]	<.0001
Sex	0.493	[0.394; 0.617]	<.0001

This analysis has been adjusted for chronological age, gender, batch, and leucocyte distribution. Hazard ratios (HR) were estimated for the association of Δage (per 5 years of age acceleration) with all-cause mortality. The HR for the "overall" dataset was 1.094 (1.022; 1.171) if only adjusted for age and sex.

Supplemental table S2. Association of cg05228408 (*CLCN6*) with all-cause mortality.

Parameter	Hazard ratio	95% CI	p-value
Discovery set (n = 864)			
CLCN6 predictor	1.211	[1.079; 1.359]	**0.0011**
Age	1.295	[1.153; 1.455]	<.0001
Sex	0.529	[0.406; 0.688]	<.0001
Validation set (n = 1000)			
CLCN6 predictor	1.114	[0.998; 1.244]	**0.055**
Age	1.237	[1.11; 1.379]	0.0001
Sex	0.54	[0.413; 0.706]	<.0001
Overall (n = 1864)			
CLCN6 predictor	1.140	[1.026; 1.266]	**0.0148**
Age	1.236	[1.114; 1.372]	0.0001
Sex	0.508	[0.406; 0.634]	<.0001

Age predictions were calculated as follows: 75.5497 - 33.4217 * (beta-value of cg05228408). This multivariate analysis has been corrected for age, sex, batch, and leukocyte distribution. Hazard ratios (HR) were estimated for the association of Δage (per 5 years of age acceleration) with all-cause mortality.

Supplemental table S3: Associations of Weidner-CpGs with all-cause mortality.

CpG ID	Gene name	FDR discovery set	Bonferroni corr. p-value discovery set	FDR validation set	Bonferroni corr. p-value validation set
cg21120249	*C9orf139*	0.00009	9.41E-05	0.08784	1
cg10917602	*HSD3B7*	0.00068	0.001366	0.19568	1
cg15804973	*MAP3K5*	0.00210	0.00629	0.00073	0.001683
cg26614073	*SCAP*	0.00622	0.024861	0.53898	1
cg03340878	*OR2B6*	0.01056	0.064096	0.07114	0.928887
cg09462576	*MRPL55*	0.01056	0.06788	0.00125	0.004994
cg05379350	*GIT1*	0.01056	0.073938	0.37529	1
cg22947000	*BCMO1*	0.01151	0.097461	0.77461	1
cg05228408	*CLCN6*	0.01151	0.113066	0.22670	1
cg23679724	*CTSZ*	0.01151	0.115081	0.57130	1
cg08598221	*SNTB1*	0.01428	0.169345	0.02503	0.200261
cg12554573	*PARP3*	0.01428	0.176708	0.56733	1
cg25268718	*PSME1*	0.01428	0.185657	0.00393	0.019673
cg15195412	*CX3CL1*	0.01462	0.204673	0.05389	0.646722
cg15297650	*TMEM163*	0.02179	0.327141	0.55054	1
cg09809672	*EDARADD*	0.02179	0.366758	0.34103	1
cg08468689	*GHDC*	0.02179	0.370472	0.30378	1
cg25256723	*F5*	0.02427	0.456139	0.99621	1
cg20654468	*LPXN*	0.02427	0.461046	0.00003	3E-05
cg23320649	*C3orf18*	0.02679	0.5549	0.19568	1
cg19724470	*CD274*	0.02679	0.574993	0.48055	1
cg26581729	*NPDC1*	0.02679	0.595022	0.01220	0.073182
cg05294455	*MYL4*	0.02679	0.616194	0.03612	0.32512
cg03224418	*SAMD10*	0.04059	0.983631	0.84482	1
cg18182399	*DES*	0.04059	1	0.89407	1
cg01820374	*LAG3*	0.04207	1	0.08784	1
cg08012287	*ACTN3*	0.04955	1	0.85844	1
cg19722847	*IPO8*	0.05261	1	0.45947	1
cg20366832	*LLGL2*	0.05496	1	0.53898	1
cg15379633	*RAB36*	0.05583	1	0.48906	1
cg22919728	*CHST13*	0.05636	1	0.85844	1
cg23124451	*CBX7*	0.06756	1	0.67505	1
cg01560871	*C10orf27*	0.06756	1	0.19568	1
cg04474832	*ABHD14B*	0.06964	1	0.89407	1
cg26927807	*BTBD2*	0.07151	1	0.85844	1
cg16363586	*BST2*	0.07227	1	0.00073	0.002202
cg00563932	*PTGDS*	0.07227	1	0.19568	1
cg02994956	*NEFH*	0.07227	1	0.84482	1
cg17471102	*FUT3*	0.07399	1	0.57130	1
cg19046959	*COL8A2*	0.07404	1	0.07114	0.996021
cg17791651	*POU3F1*	0.07869	1	0.85844	1
cg00503840	*DLX5*	0.07869	1	0.85844	1
cg05436231	*CD164L2*	0.07869	1	0.85844	1
cg03958979	*NR2E1*	0.08941	1	0.89407	1
cg26394940	*C22orf26*	0.09356	1	0.54075	1
cg18660898	*CDC42SE1*	0.10809	1	0.67505	1
cg27553955	*KCNG3*	0.11563	1	0.11575	1
cg19761273	*CSNK1D*	0.15202	1	0.02503	0.180432
cg20264732	*ESRP2*	0.16868	1	0.37529	1
cg21448423	*ACOT11*	0.17404	1	0.34121	1
cg21992250	*SLC15A3*	0.18147	1	0.95796	1
cg17861230	*PDE4C*	0.22952	1	0.55054	1
cg16386080	*CDK20*	0.22952	1	0.56733	1

cg06493994	*SCGN*	0.25725	1	0.03892	0.428147
cg17431739	*MSRB2*	0.25725	1	0.34121	1
cg17421623	*KTELC1*	0.27897	1	0.34121	1
cg08090640	*IFI35*	0.28799	1	0.63483	1
cg24178740	*FEV*	0.29605	1	0.80100	1
cg07211259	*PDCD1LG2*	0.29605	1	0.37529	1
cg25947945	*LAD1*	0.29605	1	0.89052	1
cg26610808	*BLOC1S2*	0.29605	1	0.90063	1
cg08209133	*SLC10A4*	0.29605	1	0.84482	1
cg07621046	*C10orf82*	0.29605	1	0.46745	1
cg25538571		0.30763	1	0.66243	1
cg16744741	*PRKG2*	0.34258	1	0.37529	1
cg06638433	*IGF2BP1*	0.36477	1	0.87896	1
cg01739167	*CHRNE*	0.36477	1	0.19568	1
cg25431974	*ECEL1*	0.36858	1	0.36167	1
cg24713204	*ZNF471*	0.37185	1	0.84482	1
cg21801378	*BRUNOL6*	0.37268	1	0.66243	1
cg14456683	*ZIC1*	0.37493	1	0.84482	1
cg27320127	*KCNK12*	0.41984	1	0.56733	1
cg04036898	*POMGNT1*	0.41984	1	0.84482	1
cg12883767	*SLC26A10*	0.41984	1	0.59331	1
cg04123409	*SDS*	0.41984	1	0.26414	1
cg24768561	*AGAP1*	0.49247	1	0.85844	1
cg25809905	*ITGA2B*	0.53147	1	0.50408	1
cg22736354	*NHLRC1*	0.54848	1	0.56733	1
cg13870866	*TBX20*	0.55756	1	0.22514	1
cg02844545	*GCM2*	0.59681	1	0.94806	1
cg05488632	*EPHX3*	0.60043	1	0.85844	1
cg13129046	*C10orf35*	0.61427	1	0.85844	1
cg25762706	*STMN4*	0.61427	1	0.66243	1
cg15538427	*LRRN4CL*	0.61473	1	0.41396	1
cg21870884	*GPR25*	0.61559	1	0.85844	1
cg07810156	*PDZK1IP1*	0.69495	1	0.25290	1
cg06291867	*HTR7*	0.73641	1	0.66243	1
cg22580512	*NCOR2*	0.74499	1	0.80183	1
cg02489552	*CCDC105*	0.75504	1	0.96761	1
cg02228185	*ASPA*	0.78967	1	0.85844	1
cg13870494	*MAMDC2*	0.86030	1	0.37529	1
cg13807496	*ALX4*	0.86030	1	0.85844	1
cg00489401	*FLT4*	0.88016	1	0.85844	1
cg14918082	*KCNAB3*	0.90502	1	0.48906	1
cg21296230	*GREM1*	0.91609	1	0.03854	0.385405
cg05331214	*SCN7A*	0.95150	1	0.34103	1
cg00059225	*GLRA1*	0.96415	1	0.56733	1
cg16352283	*FAM46B*	0.96415	1	0.85844	1
cg07388493	*NDUFS5*	0.96845	1	0.41136	1

Adjusted for gender and chronological age.

Supplemental table S4: Association of Hannum CpGs with all-cause mortality.

CpG ID	Gene name	FDR discovery set	Bonferroni corr. p-value discovery set	FDR validation set	Bonferroni corr. p-value validation set
cg10501210		0.00609	0.00912	0.22364	1
cg20822990	*ATP13A2*	0.00609	0.01218	0.09914	0.89224
cg07082267		0.00811	0.02434	0.74143	1
cg19283806	*CCDC102B*	0.01354	0.05415	0.89530	1
cg02046143	*IGSF9B*	0.02079	0.10891	0.98181	1
cg04416734	*ALDOA*	0.02079	0.12473	0.38152	1
cg02867102		0.02961	0.24280	0.02252	0.07453
cg09809672	*EDARADD*	0.02961	0.26091	0.37331	1
cg25428494	*HPSE*	0.02961	0.26645	0.89530	1
cg16867657	*ELOVL2*	0.03136	0.31356	0.57828	1
cg22016779	*DNER*	0.04902	0.53924	0.96173	1
ch_2_30415474F		0.06843	0.84016	0.00133	0.00133
cg22796704	*ARHGAP22*	0.06843	0.92301	0.28200	1
cg19722847	*IPO8*	0.06843	0.95806	0.50083	1
cg16054275	*F5*	0.06847	1	0.61660	1
cg07955995	*KLF14*	0.07473	1	0.89823	1
cg20426994	*KLF14*	0.07740	1	0.89823	1
cg04474832	*ABHD14B*	0.08882	1	0.95229	1
cg07583137	*CHMP4C*	0.10474	1	0.51300	1
cg06685111	*HCG18*	0.10474	1	0.89823	1
cg02085953	*ARID5A*	0.10474	1	0.74143	1
cg08234504		0.10474	1	0.96173	1
cg20052760		0.10474	1	0.02252	0.09009
cg22512670	*RPS6KA1*	0.10474	1	0.22364	1
cg14361627	*KLF14*	0.10474	1	0.89823	1
cg06874016	*NKIRAS2*	0.10958	1	0.40261	1
cg06419846	*CD248*	0.11550	1	0.89530	1
cg22285878	*KLF14*	0.11965	1	0.61660	1
cg23744638		0.13911	1	0.37331	1
cg21139312	*MSI2*	0.13975	1	0.00291	0.00582
cg14556683	*EPHX3*	0.13975	1	0.76789	1
cg08097417	*KLF14*	0.17022	1	0.89530	1
cg13001142	*STXBP5*	0.17022	1	0.96418	1
cg02650266		0.17022	1	0.49640	1
cg00748589		0.20400	1	0.95229	1
cg08540945		0.20479	1	0.58183	1
cg06493994	*SCGN*	0.24701	1	0.04387	0.30706
cg05442902	*MGC16703*	0.29585	1	0.50083	1
cg07553761	*TRIM59*	0.29585	1	0.89530	1
cg23606718	*FAM123C*	0.29585	1	0.96173	1
cg03399905	*ANKRD34C*	0.29585	1	0.89823	1
cg03473532	*MKLN1*	0.29585	1	0.50083	1
cg03032497		0.34102	1	0.95229	1
cg04400972	*TRIM45*	0.34102	1	0.89530	1
cg16419235	*PENK*	0.34636	1	0.74972	1
cg04940570	*TEAD1*	0.34636	1	0.49640	1
cg07927379	*C7orf13*	0.36531	1	0.81331	1
cg22158769	*LOC375196*	0.41298	1	0.96173	1
cg23500537		0.41489	1	0.61660	1
cg07547549	*SLC12A5*	0.45361	1	0.96173	1
cg22213242	*CD248*	0.53422	1	0.89530	1
ch_13_39564907R		0.53422	1	0.04387	0.24180
cg00481951	*SST*	0.55241	1	0.28200	1

cg19935065	*DNTT*	0.55241	1	0.28200	1
cg00486113	*PSORS1C1*	0.55241	1	0.89530	1
cg22736354	*NHLRC1*	0.57001	1	0.61544	1
cg09651136	*PKM2*	0.58038	1	0.49223	1
cg03607117	*SFMBT1*	0.62580	1	0.51256	1
cg23091758	*NRIP3*	0.62580	1	0.50083	1
cg25478614	*SST*	0.66985	1	0.28200	1
cg06639320	*FHL2*	0.67171	1	0.22364	1
cg24079702	*FHL2*	0.70653	1	0.28200	1
cg01528542		0.70760	1	0.38344	1
cg11067179	*CD248*	0.74989	1	0.61660	1
cg14692377	*SLC6A4*	0.74989	1	0.96418	1
cg22454769	*FHL2*	0.75452	1	0.55204	1
cg25410668	*RPA2*	0.81926	1	0.95229	1
cg04875128	*OTUD7A*	0.82899	1	0.89530	1
cg08415592	*APOL1*	0.83801	1	0.09432	0.75456
cg18473521	*HOXC4*	0.88294	1	0.89823	1
cg21296230	*GREM1*	0.88294	1	0.04387	0.27640

Adjusted for gender and chronological age.

Supplemental table S5: Association of Horvath-CpGs with all-cause mortality.

CpG ID	Gene name	FDR discovery set	Bonferroni corr. p-value discovery set	FDR validation set	Bonferroni corr. p-value validation set
cg15804973	*MAP3K5*	0.02243	0.02243	0.00600	0.00600
cg26614073	*SCAP*	0.03186	0.08865	0.75276	1
cg13828047	*MPI*	0.03186	0.09557	0.20056	1
cg01511567	*SSRP1*	0.05508	0.24639	0.73798	1
cg07730301	*ALDH3B1*	0.05508	0.27541	0.07808	0.48193
cg22947000	*BCMO1*	0.05792	0.34751	0.83108	1
cg21305265	*KCTD9*	0.06083	0.42583	0.83108	1
cg09646392	*TNFSF13B*	0.06773	0.55012	0.03350	0.10049
cg01459453	*SELP*	0.06773	0.68267	0.15430	1
cg26723847	*VPS26B*	0.06773	0.70428	0.54614	1
cg04121983	*CASKIN2*	0.06773	0.74506	0.45105	1
cg03330058	*ABTB1*	0.07726	0.92717	0.40559	1
cg09809672	*EDARADD*	0.09611	1	0.51108	1
cg25564800	*KPNA1*	0.09611	1	0.45105	1
cg13931228	*MPP6*	0.09914	1	0.15116	1
cg19945840	*SDF4*	0.11120	1	0.38421	1
cg10266490	*ACOT11*	0.11390	1	0.06154	0.30770
cg19724470	*CD274*	0.11390	1	0.69717	1
cg02364642	*GEFT*	0.11540	1	0.40559	1
cg24471894	*KIAA0020*	0.12334	1	0.49023	1
cg07408456	*PGLYRP2*	0.12334	1	0.45883	1
cg18055007	*DDAH2*	0.13505	1	0.08132	0.94616
cg24126851	*DCHS1*	0.15379	1	0.84853	1
cg17274064	*ERG*	0.15379	1	0.89000	1
cg01820374	*LAG3*	0.15379	1	0.24277	1
cg26824091	*GLO1*	0.15379	1	0.83108	1
cg16984944	*TBC1D23*	0.15411	1	0.38421	1
cg17324128	*RASSF4*	0.16848	1	0.08132	1
cg17686885	*TOM1L1*	0.17508	1	0.76787	1

cg19722847	*IPO8*	0.17508	1	0.69717	1
cg04528819	*KLF14*	0.19053	1	0.94664	1
cg14992253	*EIF3I*	0.19053	1	0.89967	1
cg10940099	*CD164*	0.19053	1	0.84853	1
cg14727952	*BIRC2*	0.19053	1	0.29217	1
cg12941369	*PDCD6IP*	0.19053	1	0.55865	1
cg02275294	*SOAT1*	0.19384	1	0.37686	1
cg06836772	*PRKAA2*	0.19634	1	0.77491	1
cg23124451	*CBX7*	0.19634	1	0.83108	1
cg11314684	*AKT3*	0.19634	1	0.26606	1
cg01560871	*C10orf27*	0.19634	1	0.38421	1
cg25771195	*C16orf80*	0.19634	1	0.77491	1
cg21096399	*MCAM*	0.19634	1	0.75311	1
cg04474832	*ABHD14B*	0.19634	1	0.94664	1
cg02085507	*TRIP10*	0.20824	1	0.51561	1
cg06810647	*CRAMP1L*	0.20824	1	0.69717	1
cg21950518	*IL6ST*	0.21096	1	0.95772	1
cg09418283	*PAWR*	0.21266	1	0.89000	1
cg08331960	*SLC9A3R2*	0.21266	1	0.77491	1
cg19046959	*COL8A2*	0.21551	1	0.19730	1
cg01262913	*DSCR9*	0.21989	1	0.84493	1
cg27544190	*C21orf63*	0.22630	1	0.69717	1
cg25101936	*ZBTB16*	0.24524	1	0.89967	1
cg18440048	*ZNF70*	0.24988	1	0.37686	1
cg26372517	*TFAP2E*	0.25477	1	0.68165	1
cg17338403	*SLCO3A1*	0.25477	1	0.69717	1
cg10486998	*GALR1*	0.26030	1	0.92610	1
cg26394940	*C22orf26*	0.26030	1	0.75311	1
cg00431549	*MGP*	0.26030	1	0.01323	0.02647
cg02154074	*HTRA2*	0.26057	1	0.77491	1
cg15341340	*DNASE2*	0.26057	1	0.93442	1
cg05847778	*BBS5*	0.26336	1	0.90978	1
cg04452713	*DST*	0.27613	1	0.97679	1
cg20914508	*GAP43*	0.29538	1	0.60719	1
cg25928579	*HOXB8*	0.29538	1	0.89000	1
cg16899442	*CCDC78*	0.29538	1	0.77491	1
cg22171829	*PDK4*	0.29538	1	0.37686	1
cg13302154	*MGP*	0.29538	1	0.29217	1
cg11653266	*MRPL38*	0.29598	1	0.86682	1
cg23092072	*AFF1*	0.30288	1	0.93560	1
cg08965235	*LTBP3*	0.30639	1	0.89258	1
cg21370143	*MYBPC3*	0.30639	1	0.07808	0.77389
cg04999691	*C7orf29*	0.30639	1	0.75311	1
cg19305227	*SLC28A2*	0.30639	1	0.66831	1
cg03103192	*SPATA18*	0.30946	1	0.76787	1
cg27494383	*LTK*	0.30946	1	0.45387	1
cg26456957	*PPP1R12C*	0.31908	1	0.87638	1
cg11932564	*TNFRSF13C*	0.31908	1	0.76787	1
cg19761273	*CSNK1D*	0.33114	1	0.07808	0.64336
cg14423778	*MBNL1*	0.33114	1	0.38421	1
cg14894144	*LAMA3*	0.33495	1	0.84853	1
cg14258236	*OR5V1*	0.33495	1	0.69717	1
cg14329157	*WDR69*	0.34362	1	0.76787	1
cg06117855	*CLEC3B*	0.35287	1	0.73798	1
cg25552492	*LGI3*	0.35287	1	0.53642	1
cg02827112	*SMARCAD1*	0.38276	1	0.54454	1
cg22190114	*NLRP8*	0.38364	1	0.94755	1
cg06144905	*PIPOX*	0.38364	1	0.40559	1

cg02335441	*NEK11*	0.40063	1	0.84853	1
cg13682722	*C14orf102*	0.40142	1	0.77491	1
cg27319898	*ZNF804B*	0.41046	1	0.89194	1
cg24899750	*SNRPB2*	0.41046	1	0.83108	1
cg03286783	*CASC4*	0.41046	1	0.83108	1
cg08030082	*POMC*	0.41046	1	0.97679	1
cg12946225	*HMG20B*	0.41046	1	0.91858	1
cg26845300	*SNX9*	0.41046	1	0.69717	1
cg10376763	*TNP1*	0.41046	1	0.77491	1
cg05755779	*COLEC10*	0.41046	1	0.93120	1
cg17729667	*NINL*	0.41046	1	0.77491	1
cg18031008	*MRPS21*	0.41108	1	0.73520	1
cg22289837	*CA3*	0.41850	1	0.68342	1
cg23662675	*ZMYND8*	0.41850	1	0.89000	1
cg18328933	*ABHD14B*	0.41850	1	0.53140	1
cg01656216	*ZNF438*	0.41850	1	0.77491	1
cg16419345	*ACOX1*	0.43139	1	0.89258	1
cg06738602	*PTGER2*	0.43139	1	0.88086	1
cg17099569		0.43191	1	0.77491	1
cg23941599	*FEM1C*	0.43529	1	0.79584	1
cg19346193	*BCCIP*	0.44211	1	0.58615	1
cg12351433	*LHCGR*	0.44521	1	0.40559	1
cg14723032	*PITPNM3*	0.44521	1	0.83108	1
cg14409958	*ENPP2*	0.44521	1	0.83108	1
cg06493994	*SCGN*	0.44521	1	0.10904	1
cg15974053	*HSD17B14*	0.45652	1	0.92610	1
cg16150435	*C6orf15*	0.45652	1	0.77491	1
cg22637507	*ALKBH3*	0.45652	1	0.45105	1
cg19420968	*HCRTR1*	0.45652	1	0.58615	1
cg26453588	*BIK*	0.47043	1	0.89967	1
cg06361108	*CCNF*	0.47313	1	0.89000	1
cg07291563	*GRWD1*	0.47746	1	0.31673	1
cg03891319	*ACY1*	0.50639	1	0.08132	1
cg17589341	*SLC14A1*	0.50639	1	0.83108	1
cg15185286	*AIG1*	0.50639	1	0.69717	1
cg20305610	*PDLIM5*	0.50639	1	0.75311	1
cg03270204	*DDR1*	0.50639	1	0.77491	1
cg22006386	*CATSPERG*	0.50639	1	0.77491	1
cg02217159	*KHDRBS2*	0.50639	1	0.97531	1
cg09722397	*GRIN2C*	0.50639	1	0.50111	1
cg14308452	*PRR22*	0.50639	1	0.83108	1
cg05442902	*MGC16703*	0.51174	1	0.69717	1
cg16168311	*APOA1BP*	0.51769	1	0.38421	1
cg19853760	*LGALS1*	0.51769	1	0.89258	1
cg00168942	*GJD4*	0.52814	1	0.98840	1
cg00091693	*KRT20*	0.53579	1	0.84137	1
cg14408969	*C8orf40*	0.53579	1	0.84853	1
cg17063929	*NOX4*	0.55122	1	0.46005	1
cg19706682	*LRRC50*	0.55858	1	0.51108	1
cg26003813	*PLK1*	0.55858	1	0.76787	1
cg17960516	*DOK7*	0.56253	1	0.98840	1
cg07158339	*FXN*	0.56713	1	0.92610	1
cg16744741	*PRKG2*	0.56713	1	0.57546	1
cg17285325	*TYMP*	0.57519	1	0.37686	1
cg24834740	*PPP1R16B*	0.58197	1	0.69717	1
cg27092035	*ARL10*	0.58352	1	0.94662	1
cg19514928	*TMEM56*	0.61160	1	0.45105	1
cg26005082	*MIR7-3*	0.61861	1	0.42951	1

cg14424579	*AGBL5*	0.62436	1	0.77491	1
cg05675373	*KCNC4*	0.62839	1	0.86632	1
cg21801378	*BRUNOL6*	0.62851	1	0.80710	1
cg25159610	*PLK2*	0.63603	1	0.68165	1
cg24116886	*DEFB127*	0.63609	1	0.89000	1
cg26162695	*ELAC2*	0.64156	1	0.77437	1
cg09509673	*CCR10*	0.64156	1	0.86682	1
cg21460081	*HOXB4*	0.64156	1	0.83108	1
cg13269407	*C22orf26*	0.64914	1	0.77491	1
cg25505610	*EIF3M*	0.65471	1	0.51472	1
cg04126866	*C10orf99*	0.65471	1	0.58615	1
cg25148589	*GRIA2*	0.65471	1	0.83108	1
cg18983672	*FOXE3*	0.67187	1	0.81193	1
cg03760483	*ALOX12*	0.68335	1	0.69717	1
cg04094160	*ZBTB5*	0.68510	1	0.88684	1
cg15262928	*TIMM17A*	0.68727	1	0.83108	1
cg22809047	*RPL31*	0.69058	1	0.37686	1
cg22449114	*TCF15*	0.70205	1	0.79584	1
cg14175438	*FAM3C*	0.70205	1	0.76787	1
cg09133026	*RPS6KL1*	0.70205	1	0.97679	1
cg20100381	*NAE1*	0.70494	1	0.88086	1
cg09191327	*PRDM12*	0.71651	1	0.56810	1
cg20795863	*NEU2*	0.71651	1	0.79584	1
cg20240860	*ACCS*	0.71699	1	0.89000	1
cg13547237	*C11orf68*	0.73633	1	0.77491	1
cg19478743	*ZMYND15*	0.73633	1	0.83108	1
cg00864867	*PAWR*	0.73679	1	0.96337	1
cg27169020	*BNC1*	0.73849	1	0.93560	1
cg16034652	*BTBD7*	0.73849	1	0.07808	0.67746
cg18139769	*SGCE*	0.73849	1	0.45883	1
cg08124722	*CCL7*	0.74223	1	0.89000	1
cg10865119	*C6orf122*	0.74223	1	0.74119	1
cg07498421	*CRADD*	0.74223	1	0.70676	1
cg22197830	*TXNDC15*	0.74223	1	0.77491	1
cg22901840	*DIRAS3*	0.74223	1	0.75311	1
cg14501253	*C8orf79*	0.74445	1	0.97679	1
cg16494477	*FGF18*	0.76398	1	0.97679	1
cg27413543	*SEC31A*	0.76398	1	0.97679	1
cg17853587	*NDST3*	0.76433	1	0.77491	1
cg01234063	*ST3GAL4*	0.76433	1	0.69717	1
cg13038560	*C2orf60*	0.76433	1	0.97679	1
cg24580001	*CCDC88B*	0.76462	1	0.93560	1
cg18956095	*ZHX1*	0.76462	1	0.88684	1
cg17655614	*CDH1*	0.76462	1	0.45883	1
cg25809905	*ITGA2B*	0.76799	1	0.72526	1
cg01873645	*FAM108B1*	0.77031	1	0.75276	1
cg09885951	*CENPF*	0.77031	1	0.97679	1
cg03167275	*CXADR*	0.77180	1	0.75744	1
cg16579101	*NOP2*	0.77207	1	0.80710	1
cg22736354	*NHLRC1*	0.78228	1	0.76787	1
cg01485645	*MLLT6*	0.78543	1	0.93560	1
cg23517605	*TUBB2B*	0.79843	1	0.99377	1
cg03019000	*TEX264*	0.80002	1	0.05279	0.21115
cg25070637	*SDC2*	0.80008	1	0.76787	1
cg11388238	*KCTD18*	0.80030	1	0.37686	1
cg06993413	*DPP8*	0.80064	1	0.07808	0.78075
cg08090772	*ADHFE1*	0.80254	1	0.56676	1
cg12985418	*MIB1*	0.80254	1	0.83848	1

cg08370996	*NR2F2*	0.80428	1	0.84399	1
cg19008809	*SFMBT1*	0.80695	1	0.83108	1
cg10045881	*CHI3L2*	0.81962	1	0.29217	1
cg23180365	*GLB1*	0.82543	1	0.92610	1
cg12373771	*CECR6*	0.83203	1	0.77491	1
cg26620959	*SYNE1*	0.83203	1	0.97679	1
cg27377450		0.83203	1	0.75311	1
cg09118625	*DIRAS3*	0.83203	1	0.69717	1
cg01584473	*MUC17*	0.83203	1	0.77491	1
cg02332492	*C8G*	0.83203	1	0.88684	1
cg13129046	*C10orf35*	0.83203	1	0.89000	1
cg14658362	*RBPMS*	0.83203	1	0.94664	1
cg09722555	*CCL27*	0.83203	1	0.89258	1
cg19167673	*PDGFB*	0.83203	1	0.75276	1
cg25683012	*BAZ2A*	0.83203	1	0.77491	1
cg18180783	*MYOZ1*	0.83203	1	0.89000	1
cg02479575	*MIR7-3*	0.83203	1	0.89000	1
cg09019938	*PRKG1*	0.83203	1	0.89000	1
cg07285276	*RAPGEF1*	0.83420	1	0.89967	1
cg21870884	*GPR25*	0.83420	1	0.90657	1
cg08413469	*DEPDC1*	0.83420	1	0.38421	1
cg11025793	*IER2*	0.85153	1	0.18635	1
cg15703512	*C16orf65*	0.86317	1	0.34299	1
cg24450312	*RASSF5*	0.86317	1	0.98781	1
cg10345936	*SLC36A2*	0.86317	1	0.89000	1
cg13899108	*PDE4C*	0.86317	1	0.80007	1
cg05365729	*LOXL2*	0.86317	1	0.93560	1
cg24081819	*EPHX2*	0.86317	1	0.69717	1
cg20524216	*C3orf75*	0.86317	1	0.83108	1
cg00374717	*ARSG*	0.86317	1	0.58267	1
cg24254120	*RFC3*	0.86317	1	0.97679	1
cg27015931	*C16orf65*	0.86317	1	0.78704	1
cg14597908	*GNASAS*	0.86317	1	0.45883	1
cg15661409	*C14orf105*	0.86317	1	0.97679	1
cg13836627	*TJP1*	0.86317	1	0.92610	1
cg26297688	*C12orf23*	0.86317	1	0.76787	1
cg20295671	*YPEL1*	0.86317	1	0.73798	1
cg16408394	*RXRA*	0.86317	1	0.94664	1
cg03682823	*SGCE*	0.86317	1	0.37686	1
cg25781123	*THUMPD3*	0.86317	1	0.89258	1
cg15547534	*C7orf47*	0.86317	1	0.83108	1
cg05903609	*PRPF8*	0.86317	1	0.37686	1
cg18984151	*C3orf75*	0.86317	1	0.88684	1
cg19044674	*LEPRE1*	0.86317	1	0.97679	1
cg13975369	*TSGA14*	0.86317	1	0.83108	1
cg02331561	*ABCA17P*	0.86485	1	0.75311	1
cg09785172	*WFS1*	0.86485	1	0.89000	1
cg25166896	*C22orf25*	0.86485	1	0.94664	1
cg00436603	*CYP2E1*	0.86683	1	0.69717	1
cg06044899	*TMSL3*	0.86683	1	0.45105	1
cg05960024	*CLOCK*	0.86683	1	0.89000	1
cg01027805	*ZNF219*	0.86683	1	0.83108	1
cg16358826	*GABRA4*	0.87269	1	0.88684	1
cg23786576	*ATPAF1*	0.87269	1	0.83108	1
cg04268405	*CHST3*	0.87269	1	0.77491	1
cg20828084	*KIAA1199*	0.87269	1	0.93560	1
cg06926735	*UBE2V1*	0.87269	1	0.83108	1
cg05590257	*PLD6*	0.87269	1	0.79584	1

cg16241714	*CEBPD*	0.87366	1	0.92597	1
cg07663789	*NPR3*	0.87366	1	0.69717	1
cg19569684	*MGC29506*	0.87366	1	0.94664	1
cg04005032	*OSBPL10*	0.87366	1	0.93120	1
cg10281002	*TBX5*	0.87366	1	0.88684	1
cg05294243	*KLK13*	0.87366	1	0.40559	1
cg01027739	*DOLPP1*	0.87366	1	0.69717	1
cg13319175	*CAPZB*	0.88378	1	0.96884	1
cg09869858	*P11*	0.88378	1	0.77491	1
cg02489552	*CCDC105*	0.88378	1	0.98289	1
cg02047577	*UCKL1AS*	0.88378	1	0.92597	1
cg12413566	*XIRP1*	0.88378	1	0.97679	1
cg21395782	*NDUFA13*	0.88686	1	0.89000	1
cg12830694	*PPP1R14A*	0.91426	1	0.75311	1
cg01353448	*C7orf16*	0.91426	1	0.83108	1
cg06462291	*NT5DC3*	0.91426	1	0.76787	1
cg06513075	*NAT10*	0.91426	1	0.77491	1
cg07595943	*ADAD2*	0.91787	1	0.77491	1
cg01407797	*CCDC117*	0.91787	1	0.73798	1
cg06952310	*NCAN*	0.91819	1	0.94664	1
cg20692569	*FZD9*	0.91846	1	0.97679	1
cg26043391	*FBXO28*	0.92017	1	0.83108	1
cg06121469	*SPG11*	0.92387	1	0.93560	1
cg03947362	*C2orf60*	0.92872	1	0.96337	1
cg08771731	*LOC285696*	0.92872	1	0.93560	1
cg08251036		0.92872	1	0.89000	1
cg02972551	*KDM3A*	0.93453	1	0.93560	1
cg24058132	*GALC*	0.94642	1	0.83108	1
cg24888049	*FES*	0.96322	1	0.89000	1
cg19692710	*DNAJB13*	0.96824	1	0.83108	1
cg07849904	*MN1*	0.97089	1	0.84625	1
cg03578041	*LARP6*	0.97089	1	0.81661	1
cg20761322	*CIB2*	0.97089	1	0.83108	1
cg05250458	*ZNF177*	0.97390	1	0.83108	1
cg25411725	*SLC22A13*	0.99182	1	0.78363	1
cg15381769	*PTPRK*	0.99182	1	0.85533	1
cg25657834	*NTSR2*	0.99182	1	0.45105	1
cg13216057	*DKK3*	0.99322	1	0.83108	1
cg21211748	*E2F2*	0.99322	1	0.93120	1
cg22568540	*NCRNA00181*	0.99322	1	0.98781	1
cg06557358	*TMEM132E*	0.99322	1	0.94664	1
cg26842024	*KLF2*	0.99322	1	0.86030	1
cg01570885	*FAM50B*	0.99322	1	0.69717	1
cg07455279	*NDUFA3*	0.99322	1	0.94664	1
cg04431054	*PRRC1*	0.99322	1	0.45883	1
cg02654291	*C9orf64*	0.99322	1	0.83108	1
cg26045434	*HR*	0.99322	1	0.99096	1
cg00945507	*SEC61G*	0.99322	1	0.24277	1
cg24262469	*TIPARP*	0.99322	1	0.51472	1
cg22920873	*C7orf55*	0.99322	1	0.29217	1
cg20947775	*SCD5*	0.99322	1	0.98781	1
cg09441152	*PQLC1*	0.99322	1	0.83108	1
cg15988232	*CSPG5*	0.99322	1	0.83848	1
cg13460409	*DSCR6*	0.99322	1	0.83108	1
cg06688848	*RSPRY1*	0.99322	1	0.83108	1
cg13854874	*CHAF1B*	0.99322	1	0.37686	1
cg02071305	*VPS18*	0.99322	1	0.97679	1
cg18573383	*KCNC2*	0.99322	1	0.84493	1

cg04836038	*DOCK9*	0.99541	1	0.98781	1
cg00075967	*STRA6*	0.99841	1	0.83108	1
cg12768605	*LYPD5*	0.99841	1	0.92597	1
cg27016307	*HRC*	0.99841	1	0.84399	1
cg22613010	*CLCN2*	0.99841	1	0.45105	1
cg05921699	*CD79A*	0.99841	1	0.77491	1
cg01644850	*ZNF551*	0.99841	1	0.77491	1
cg14163776	*ACAP2*	0.99841	1	0.97679	1
cg10523019	*RHBDD1*	0.99841	1	0.97679	1
cg08186124	*LZTFL1*	0.99841	1	0.76787	1
cg07337598	*ANXA9*	0.99841	1	0.77491	1
cg11299964	*MAPKAP1*	0.99918	1	0.83108	1
cg02388150	*SFRP1*	0.99918	1	0.83108	1
cg22432269	*CYFIP1*	0.99918	1	0.38421	1
cg19273182	*PAPOLG*	0.99918	1	0.97679	1
cg03588357	*GPR68*	0.99918	1	0.77491	1
cg07770222	*C8orf31*	0.99918	1	0.56676	1
cg01968178	*REEP1*	0.99918	1	0.40144	1
cg27202708	*C1orf65*	0.99918	1	0.89000	1
cg04084157	*VGF*	0.99918	1	0.97679	1
cg03565323	*ZNF287*	0.99918	1	0.76787	1
cg07388493	*NDUFS5*	0.99918	1	0.63560	1
cg16547529	*KLHL35*	0.99918	1	0.60350	1
cg17408647	*C7orf44*	0.99918	1	0.89967	1
cg21378206	*IL1F5*	0.99918	1	0.90978	1
cg22679120	*SNX8*	0.99918	1	0.80849	1
cg14060828	*PTH2*	0.99918	1	0.84853	1
cg10377274	*PATE1*	0.99918	1	0.89967	1
cg14654875	*NAT15*	0.99918	1	0.56676	1
cg10920957	*JPH3*	0.99918	1	0.51523	1
cg02580606	*KRT33B*	0.99918	1	0.92597	1
cg08434234	*DGKI*	0.99918	1	0.98781	1
cg20999813	*USP10*	0.99918	1	0.83108	1
cg12616277	*ESYT3*	0.99918	1	0.83108	1

Adjusted for gender and chronological age.

Supplemental table S6: Overlap of significant CpGs in discovery and validation set.

	Weidner	Hannum	Horvath
total CpGs	99 (61 hypo; 38 hyper)	71 (31 hypo; 40 hyper)	353 (186 hypo; 167 hyper)
significant CpGs in discovery set	27 (26 hypo; 1 hyper[§])	11 (10 hypo; 1 hyper[#])	3 (3 hypo; 0 hyper)
significant CpGs in validation set	11 (9 hypo; 2 hyper)	7 (4 hypo; 3 hyper)	3 (3 hypo; 0 hyer)
overlap	7 (7 hypo; 0 hyper[%])	1 (1 hypo; 0 hyper)	1 (1 hypo; 0 hyper)
p-value for overlap	0.0073	0.414	0.025

This table depicts the number of CpGs of the three age-predictors (with numbers of CpGs that reveal age-associated hypo- or hyper-methylation upon aging). The number of CpGs that reached statistical significance in the training and validation set of the ESTHER cohort are indicated. We determined the overlap of these two independent datasets and estimated the significance (p-value) by hypergeometric distribution. This measure indicates if the same CpGs are associated with life-expectancy in different datasets.

Furthermore, the probability was estimated for the distribution of hypo- and hypermethylated CpGs (hypogeometric distribution; [§]p-value: $3.3*10^{-6}$; [%]p-value: 0.029; [#]p-value = 0.0007). These p-values were calculated for each of the two datasets and the overlap separately.

References of supplemental data

1. Saum KU, Dieffenbach AK, Muller H, Holleczek B, Hauer K, Brenner H. Frailty prevalence and 10-year survival in community-dwelling older adults: results from the ESTHER cohort study. *Eur J Epidemiol* 2014;29:171-179.
2. Perna L, Zhang Y, Mons U, Holleczek B, Saum KU, Brenner H. Epigenetic age acceleration predicts cancer, cardiovascular, and all-cause mortality in a German case cohort. *Clin Epigenetics* 2016;8:64.
3. Miller SA, Dykes DD, Polesky HF. A simple salting out procedure for extracting DNA from human nucleated cells. *Nucleic Acids Res* 1988;16:1215.
4. Florath I, Butterbach K, Muller H, Bewerunge-Hudler M, Brenner H. Cross-sectional and longitudinal changes in DNA methylation with age: an epigenome-wide analysis revealing over 60 novel age-associated CpG sites. *Hum Mol Genet* 2014;23:1186-1201.
5. Hannum G, Guinney J, Zhao L, Zhang L, Hughes G, et al. Genome-wide Methylation Profiles Reveal Quantitative Views of Human Aging Rates. *Mol Cell* 2013;49:459-367.
6. Horvath S. DNA methylation age of human tissues and cell types. *Genome Biol* 2013;14:R115.
7. Lin Q, Weidner CI, Costa IG, Marioni RE, Ferreira MR, Deary IJ, Wagner W. DNA methylation levels at individual age-associated CpG sites can be indicative for life expectancy. *Aging (Albany NY)* 2016;8:394-401.
8. Weidner CI, Lin Q, Koch CM, Eisele L, Beier F, et al. Aging of blood can be tracked by DNA methylation changes at just three CpG sites. *Genome Biol* 2014;15:R24.
9. Kulathinal S, Karvanen J, Saarela O, Kuulasmaa K. Case-cohort design in practice - experiences from the MORGAM Project. *Epidemiol Perspect Innov* 2007;4:15.
10. Zhang Y, Breitling LP, Balavarca Y, Holleczek B, Schottker B, Brenner H. Comparison and combination of blood DNA methylation at smoking-associated genes and at lung cancer-related genes in prediction of lung cancer mortality. *Int J Cancer* 2016;139:2482-2492.
11. Houseman EA, Molitor J, Marsit CJ. Reference-free cell mixture adjustments in analysis of DNA methylation data. *Bioinformatics* 2014;30:1431-1439.
